# Minot A. Steele

**Published:** 1913-04

**Authors:** 


Minot A. Steele, Albany, 1890, died in Newport, R. I., Feb. 17, from erysipelas following a dog bite. He was 45 years old.

